# Prognostic value of new-onset right bundle-branch block in acute myocardial infarction patients: a systematic review and meta-analysis

**DOI:** 10.7717/peerj.4497

**Published:** 2018-03-12

**Authors:** Juntao Wang, Hongxing Luo, Chunling Kong, Shujuan Dong, Jingchao Li, Haijia Yu, Yingjie Chu

**Affiliations:** 1 Department of Cardiology, Zhengzhou University People’s Hospital, Zhengzhou, China; 2 Department of Cardiology, Henan Province People’s Hospital, Zhengzhou, China

**Keywords:** Myocardial infarction, Bundle-branch block, Prognosis, Meta-analysis

## Abstract

**Background:**

Patients with acute myocardial infarction (AMI) and bundle-branch block have poor prognoses. The new European Society of Cardiology guideline suggests a primary percutaneous coronary intervention strategy when persistent ischemic symptoms occur in patients with persistent ischemic symptoms and right bundle-branch block (RBBB), but the level of evidence is not high. In fact, the presence of RBBB may lead to the misdiagnosis of transmural ischemia and mask the early diagnosis of ST-elevation myocardial infarction. Moreover, new-onset RBBB is occasionally caused by AMI. Our study aims to investigate the prognostic value of new-onset RBBB in AMI.

**Methods and Results:**

We conducted a meta-analysis of studies to evaluate the prognostic value of RBBB in AMI patients. Of 914 primary records, five studies and 874 MI patients were included for meta-analysis. Compared with previous RBBB, AMI patients with new-onset RBBB had a higher risk of long-term mortality (RR, 1.66, 95% CI [1.31–2.09], *I*^2^ = 0.0%, *p* = 0.000, *n* = 2), ventricular arrhythmia (RR, 4.86, 95% CI [2.10–11.27], *I*^2^ = 0.0%, *p* = 0.000, *n* = 3), and cardiogenic shock (RR, 2.76, 95% CI [1.66–4.59], *I*^2^ = 0.0%, *p* = 0.000, *n* = 3), but a lower risk of heart failure (RR, 0.66, 95% CI [0.52–0.85], *I*^2^ = 2.50%, *p* = 0.001, *n* = 4). Compared with AMI patients with new-onset permanent RBBB, patients with new-onset transient RBBB had a lower risk of short-term mortality (RR, 0.20, 95% CI [0.11–0.37], *I*^2^ = 44.1%, *p* = 0.000, *n* = 4).

**Conclusion:**

New-onset RBBB is likely to increase long-term mortality, ventricular arrhythmia, and cardiogenic shock, but not heart failure in AMI patients. AMI patients with new-onset transient RBBB have a lower risk of short-term mortality than those with new-onset permanent RBBB. Revascularization therapies should be considered when persistent ischemic symptoms occur in patients with RBBB, especially new-onset RBBB.

## Introduction

Acute myocardial infarction (AMI) patients with right bundle-branch block (RBBB) have worse prognosis than those without RBBB, yet investigators of these studies have not compared the effects of new-onset RBBB with previous RBBB (Bhalli et al., 2009; Widimsky et al., 2012). A recent systematic review (Hazem et al., 2014) has shown that patients with RBBB and AMI are at over two-fold higher risks of all-cause mortality in 30-day follow-up compared to those without bundle-branch block (BBB). Furthermore, for patients with myocardial infarction, several other studies have reported a positive association between RBBB and all-cause mortality (Kleemann et al., 2008; Widimsky et al., 2012; Wong et al., 2006), whereas others have reported no associations (Archbold et al., 1998; Juarez-Herrera & Jerjes-Sanchez, 2013).

Considering that the blood supply of the right bundle-branch is mainly provided by left anterior descending artery (LAD) or the proximal septal perforator branch separated from LAD (Stambler, Rahimtoola & Ellenbogen, 2007), new-onset RBBB may indicate the proximal occlusion of the LAD and large infarction, thus may result in severe heart failure, complete AV block, malignant arrhythmias, and a high mortality. The classification of RBBB according to onset time, duration, and its association with fascicular block is of clinical importance (Hindman et al., 1978; Lie et al., 1974; Ricou et al., 1991). Previous studies of thrombolytic therapies have demonstrated reduction in infarct size (Kloner et al., 1983; Braunwald, 1987), improvements in late ventricular morphology and function (White et al., 1987), and reduction in mortality (Yusuf et al., 1990; Grines & DeMaria, 1990; Nicod, Zimmermann & Scherrer, 1993; Fibrinolytic Therapy Trialists, 1994). Moreover, some studies have reported the relationship between the reversibility of conduction disturbances and coronary reperfusion (Roth et al., 1993; Wiseman, Ohman & Wharton, 1989), which suggests that reperfusion therapy may prevent the appearance or limit the duration of BBB. Thus, it is probable that the current reperfusion therapy has changed the overall incidence and significance of RBBB in AMI. Therefore, it is reasonable to reanalyze and review its significance in the reperfusion therapy era. Moreover, given the poor prognosis of AMI patients with RBBB and the difficulty of determining the transmurality of an infarct in the setting of RBBB, the new European Society of Cardiology (ESC) guideline (Ibanez et al., 2017) suggests a primary percutaneous coronary intervention (PCI) strategy when persistent ischemic symptoms occur in patients with RBBB, but the level of evidence for revascularization is not high. Of note, some ambiguity exists as the Widimsky’s paper referenced in the ESC guideline has mixed new-onset RBBB and presumed RBBB in their cohort.

Accordingly, we conducted this meta-analysis to assess the prognostic value of both transient and permanent new-onset RBBB in AMI patients regarding mortality and major adverse cardiovascular events (arrhythmia, heart failure, cardiogenic shock, reinfarction, etc.).

## Methods and Analysis

Our study protocol has been registered in PROSPERO website (PROSPERO Registration Number: CRD42017070425) and our study complied with the PRISMA statement (Hutton et al., 2015). The eligibility criteria and search strategies have been illustrated in our previous article (Wang et al., 2017). New-onset RBBB was classified as transient when it disappeared at the time of hospital discharge and as permanent when the patient either died or was discharged with RBBB. According to the 2016 ESC heart failure guidelines (Ponikowski et al., 2016) and the included studies, we defined HF as a reduced cardiac output and/or elevated intracardiac pressures at rest or during stress. Cardiogenic shock was defined as hemodynamic instability in spite of increasing doses of catecholamines and/or mechanical circulatory support with critical hypoperfusion of target organs.

### Database searches

On June 13, 2017, we (JTW & HXL) searched PubMed, EMBASE, Ovid, Cochrane Library, and Web of Science with terms (acute myocardial infarction OR AMI OR Acute heart infarction) AND (bundle-branch block OR bundle-branch block OR BBB) AND (prognosis OR survival analysis OR mortality OR death OR outcome OR follow-up). Reference lists of related reviews and eligible studies were manually searched to identify potential studies for inclusion.

### Eligibility criteria

Original articles that reported the prognosis of AMI patients with new-onset RBBB were initially included. Then we excluded articles which did not report the comparison of new-onset and previous RBBB. Studies of missing values were also excluded.

### Study records

**Figure 1:**
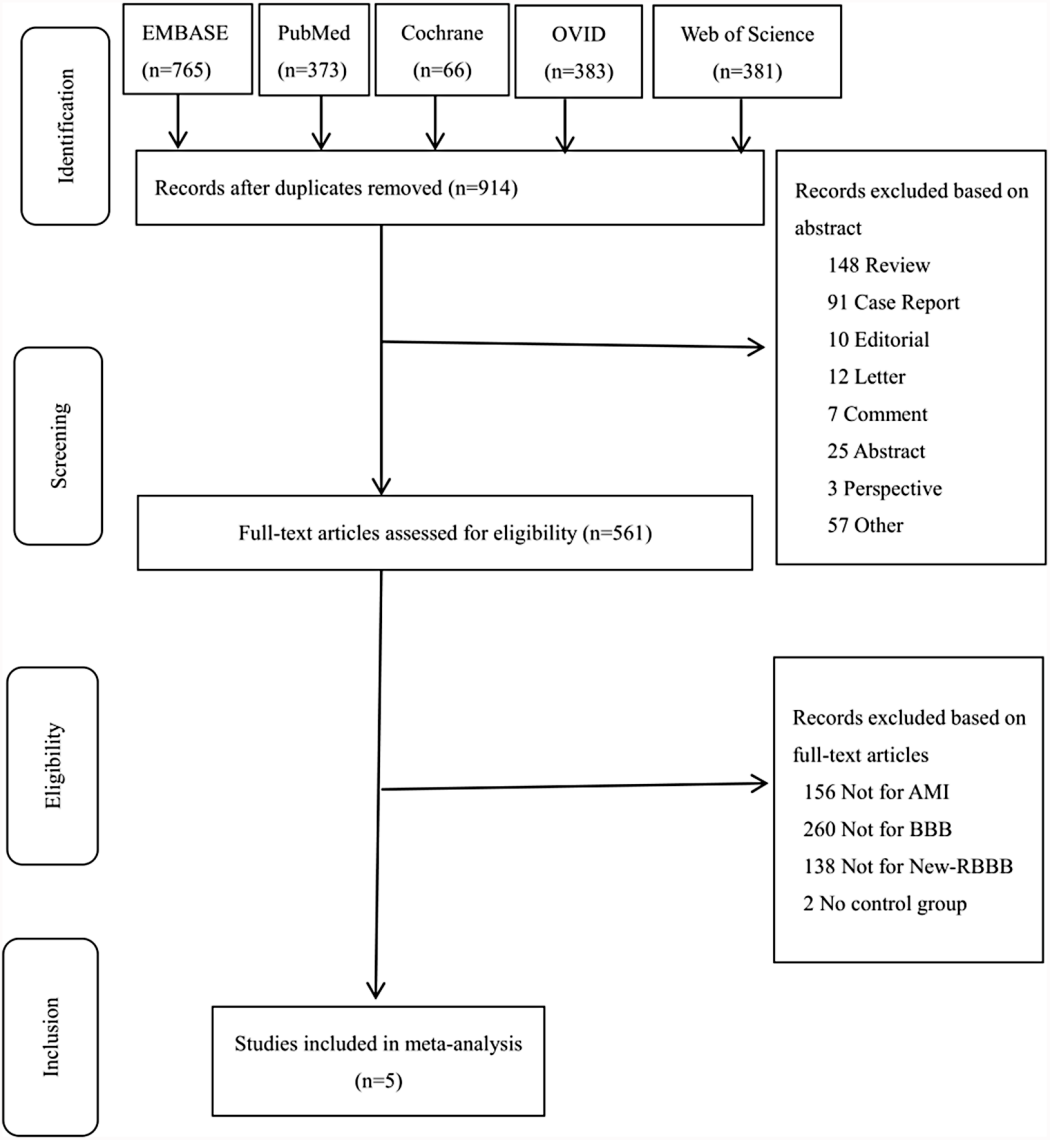
PRISMA flowchart of study selection.

Retrieved literature were exported from online databases and imported to NoteExpress Management software (v3.2.0.6941) to remove duplicates. Then items were uploaded to the Covidence website (https://www.covidence.org/). Two reviewers (JTW & CLK) independently screened the items to determine study eligibility online according to the patient-intervention-control-outcomes criteria. Reasons for exclusion were logged in a table. A third reviewer (HXL) was delegated to resolve the discrepancies through discussion. We used a PRISMA flow diagram (Fig. 1) to record the information of screening.

### Risk of bias assessment

We assessed the quality of included studies using the risk of bias tool of Newcastle–Ottawa Quality Assessment (Mcpheeters et al., 2012). Funnel plots and Egger’s test were made to assess publication bias.

### Statistical analysis

Results were expressed as relative risk (RR) with 95% CI for each study. A pooled effect size was calculated using a random-effects model. Heterogeneity was assessed using Q and *I*^2^ statistics. Sensitivity analysis was performed through the influence analysis method. All statistical analyses were performed using STATA 14.0. Statistical significance was defined as *p* < 0.05.

## Results

### Study characteristics

Table 1 shows the basic characteristics of the eligible studies. Of the five studies, three were conducted in Spain, one in the United States, and one in Japan. The diagnosis of RBBB was based on electrocardiogram in all studies. The total number of participants included in this meta-analysis was 874. The study population in five studies consisted of both men and women. The studies varied with regards to follow-up duration (3.7 day–1 year) and controlled variables in the multivariate models. Four studies reported short-term mortality, four ventricular arrhythmia, and four mechanical complications and heart failure as clinical outcomes.

**Table 1:** Clinical characteristics.

First author (year)	Study design	Country or region	Definition for RBBB	Types of included RBBB	Study participants	Study period	Sample size	RBBB			
								New-onset	Transient	Permanent	Previous
Gann et al. (1975)	Retrospective	USA	rsR′, rSR′ or qR complex ≥0.12 s duration in the right precordial leads	RBBB, with either normal axis, left or right axis deviation	Patients with acute myocardial infarction	1971–1972	75	35			40
Melgarejo-Moreno et al. (1997)	Prospective	Spain	RBBB was defined by using standard ECG criteria; a QRS duration of ≥120 ms was required	Compared with complete AVB or not	Patients consecutively diagnosed with acute myocardial infarction	1992.6–1994.1	96	51	25	26	45
Vivas et al. (2010)	Cohort	Spain	BBB was present when the QRS duration was ≥120 ms. RBBB was present when the secondary *R* wave (*R*′) in *V*_1_ and a wide *S* wave in leads *V*_5_ to *V*_6_ were detected	Compared with complete AVB or not	Consecutive patients with STEMI undergoing primary PCI	2004.1–2008.6	119	92	47	42	27
Melgarejo-Moreno et al. (2015)	Prospective	Spain	Conduction disturbances were defined using standard electrocardiographic criteria	NA	Patients with acute MI	1998.1–2008.1	465	212	137	75	253
Iwasaki et al. (2009)	Retrospective	Japan	(1) a QRS duration ≥120 ms, (2) the presence of an rSR′ pattern of QRS in lead *V*_1_, (3) a PQ interval >120 ms, and (4) a *S* wave in lead I and either lead *V*_5_ or *V*_6_	NA	Acute anterior or inferior myocardial infarction within 48 h after the onset of symptoms	1997.1.1–2006.12.31	119	99	58	41	20

Age		Male		Previous angina		Previous MI		Diabetes		Hypertension		New			Previous			LVEF, *n* (%)		Follow up
New-onset	Previous	New	Previous	New	Previous	New	Previous	New	Previous	New	Previous	I	II	III	I	II	III	New	Previous	
73.6	72.4	21	38																	3.7 day
65 ± 10	68 ± 9	30	37	13	26	8	15	14	24	20	22		27(>I)			18(>I)		20 (42 ± 11)	27 (39 ± 120)	One year
68 ± 13/68 ± 12	72 ± 9	69	22			16	5	33	11	54	8	33	59 (≥II)		25	2 (≥II)				In-hospital
69.5 (60.5–76.7)	73.5 (67.7–78.5)	159	209			27	79	84	123	100	161									One to seven years
67 ± 12		106				26		28		57										In-hospital

**Notes:**

Summary of clinical characteristics of eligible studies.

RBBB, right bundle-branch block; PCI, percutaneous coronary intervention; STEMI, ST-elevation myocardial infarction; LVEF, left ventricular ejection fraction.

Assessment of study quality yielded a score of six to seven on the scale of the nine-point scoring system for each study. The scores of included studies were recorded in Table 2. Most included studies were high-quality.

**Table 2:** Quality assessment.

First author (year)	Selection				Comparability	Outcome			Total score
	No. (1)	No. (2)	No. (3)	No. (4)	No. (1)	No. (1)	No. (2)	No. (3)	
Gann et al. (1975)	*	*			**	*	*		6
Melgarejo-Moreno et al. (1997)	*	*			**	*	*	*	7
Vivas et al. (2010)	*	*			**	*	*	*	7
Melgarejo-Moreno et al. (2015)	*	*			**	*	*	*	7
Iwasaki et al. (2009)	*	*			**	*	*		6

**Note:**

Newcastle–Ottawa quality assessment of included studies.

### Outcomes

**Figure 2:**
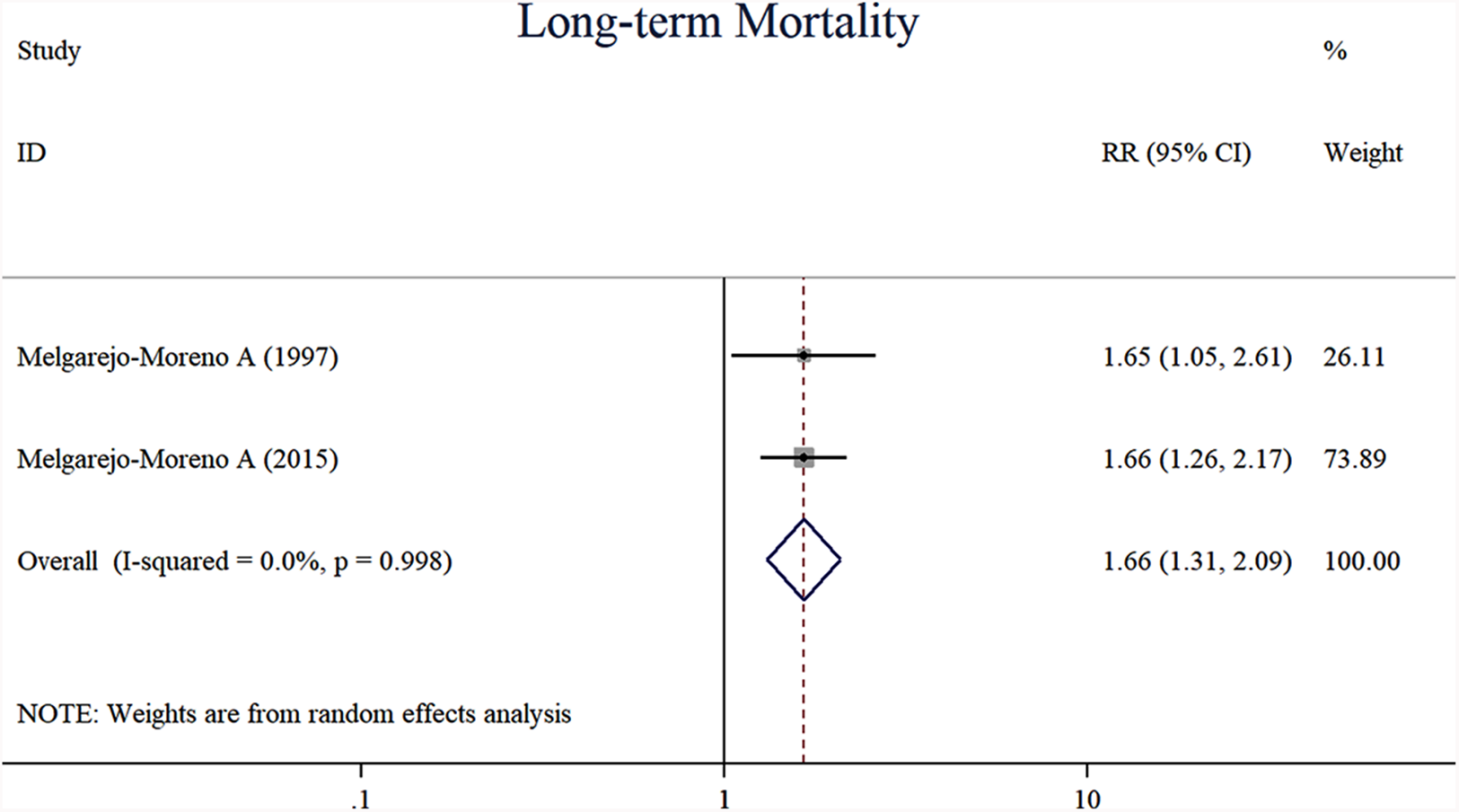
Forest plots of stratified analyses for long-time mortality.

**Figure 3:**
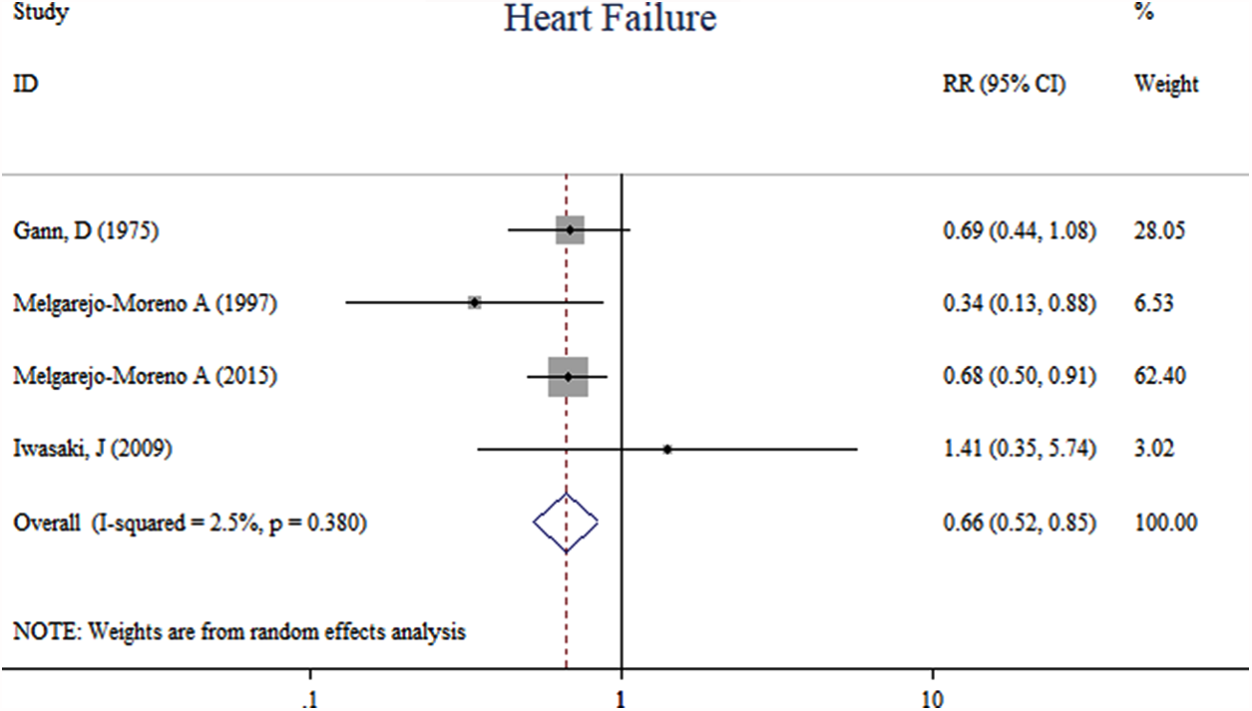
Forest plots of stratified analyses for heart failure.

**Figure 4:**
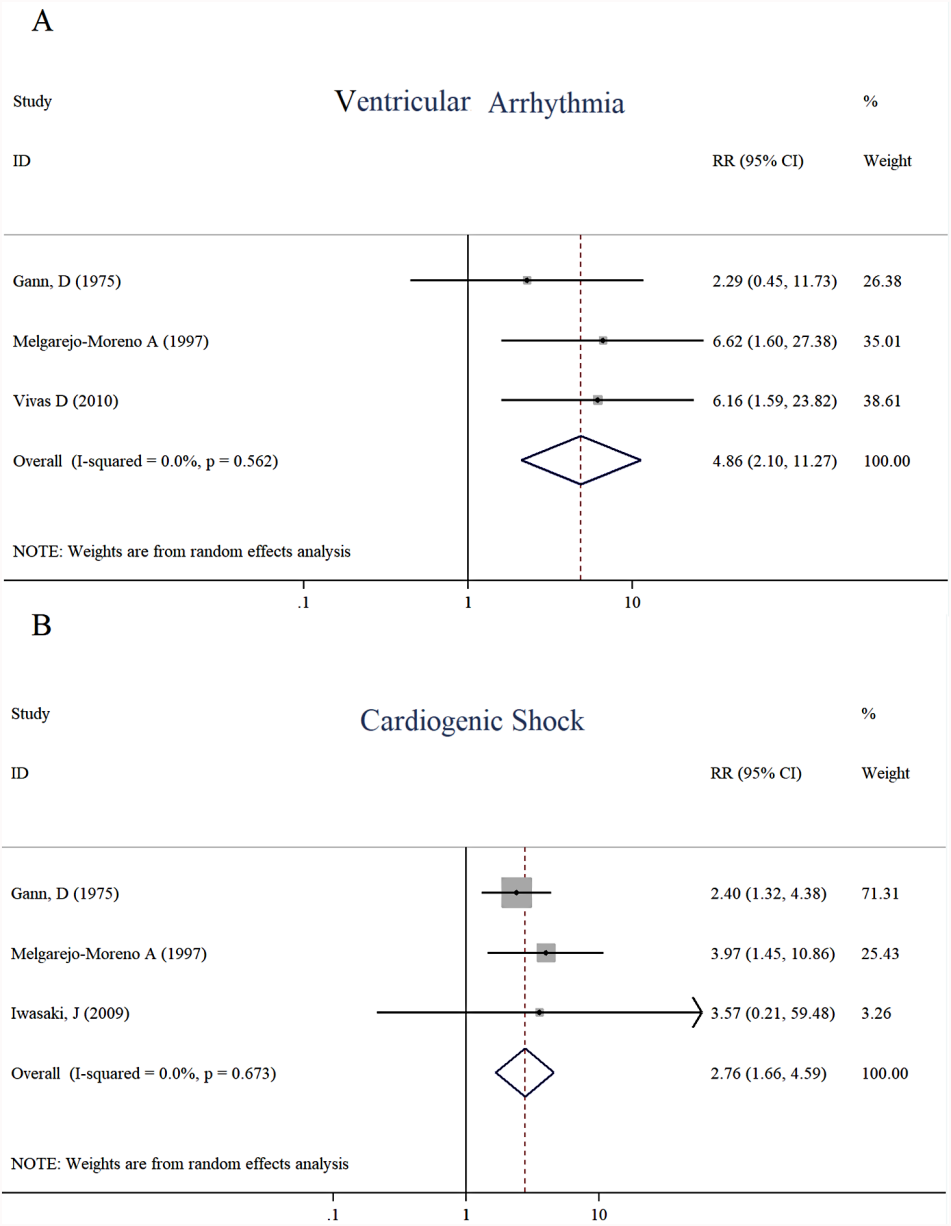
Forest plots of stratified analyses for ventricular arrhythmia (A) and cardiogenic shock (B).

Compared with previous RBBB, AMI patients with new-onset RBBB were likely associated with a higher risk of long-term (one-year) mortality (RR, 1.66, 95% CI [1.31–2.09], *I*^2^ = 0.0%, *p* = 0.000, *n* = 2), (heterogeneity, *I*^2^ = 0.0%, *p* = 0.998) (Fig. 2). The incidence of heart failure was higher in the previous RBBB group (RR, 0.66, 95% CI [0.52–0.85], *I*^2^ = 2.50%, *p* = 0.001, *n* = 4) (heterogeneity, *I*^2^ =2.5%, *p* = 0.380) (Fig. 3). New-onset RBBB group had higher risks of ventricular arrhythmia (RR, 4.86, 95% CI [2.10–11.27], *I*^2^ = 0.0%, *p* = 0.000, *n* = 3) (heterogeneity, *I*^2^ = 0.0%, *p* = 0.562) (Fig. 4A), and cardiogenic shock (RR, 2.76, 95% CI [1.66–4.59], *I*^2^ = 0.0%, *p* = 0.000, *n* = 3) (heterogeneity, *I*^2^ = 0.0%, *p* = 0.673) (Fig. 4B).

**Figure 5:**
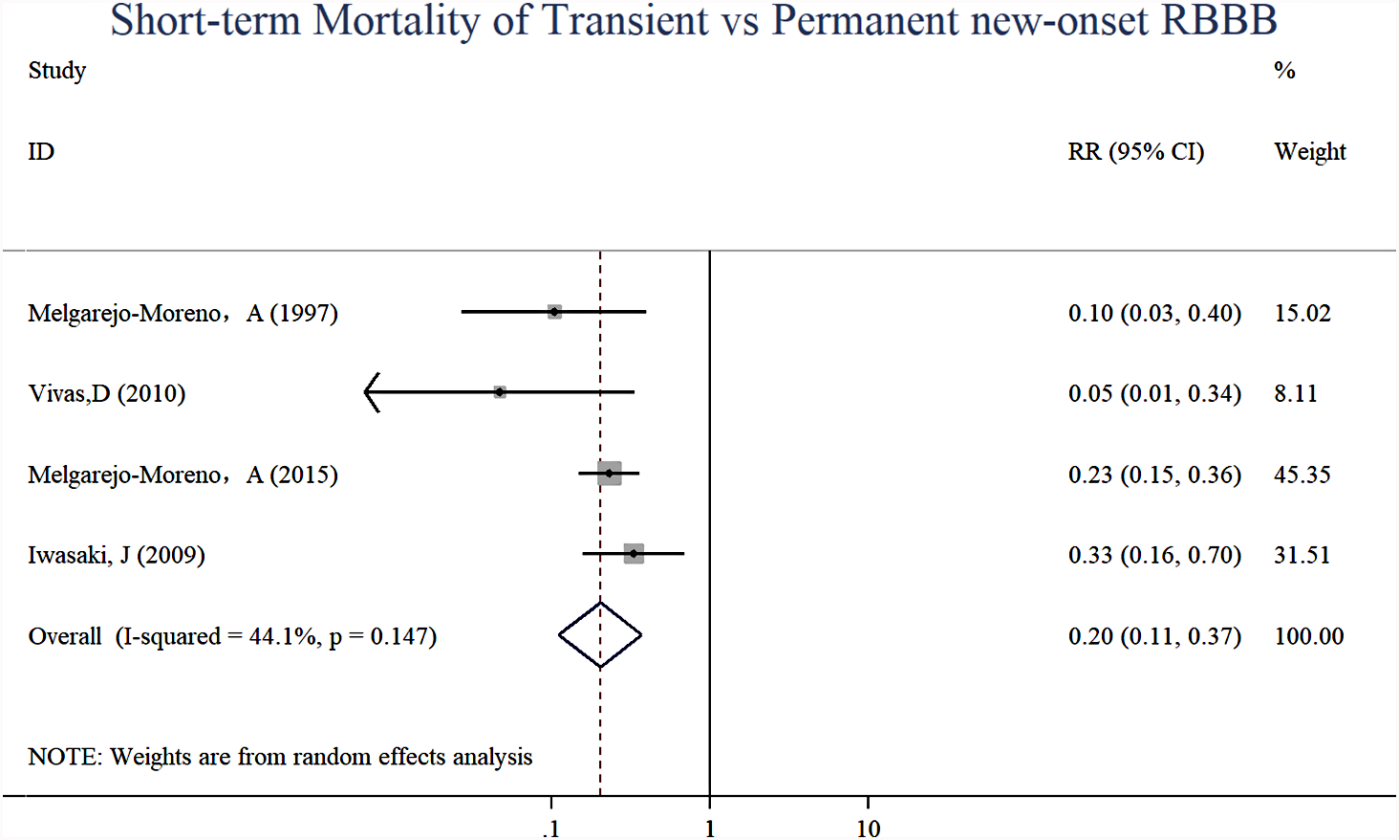
Forest plots of stratified analyses for short-time mortality (transient vs. permanent new-onset RBBB).

Compared with new-onset permanent RBBB, AMI patients with new-onset transient RBBB had a lower risk of short-term (in-hospital/30-day) mortality (RR, 0.20, 95% CI [0.11–0.37], *I*^2^ = 44.1%, *p* = 0.000, *n* = 4) (heterogeneity, *I*^2^ = 44.1%, *p* = 0.147) (Fig. 5).

**Figure 6:**
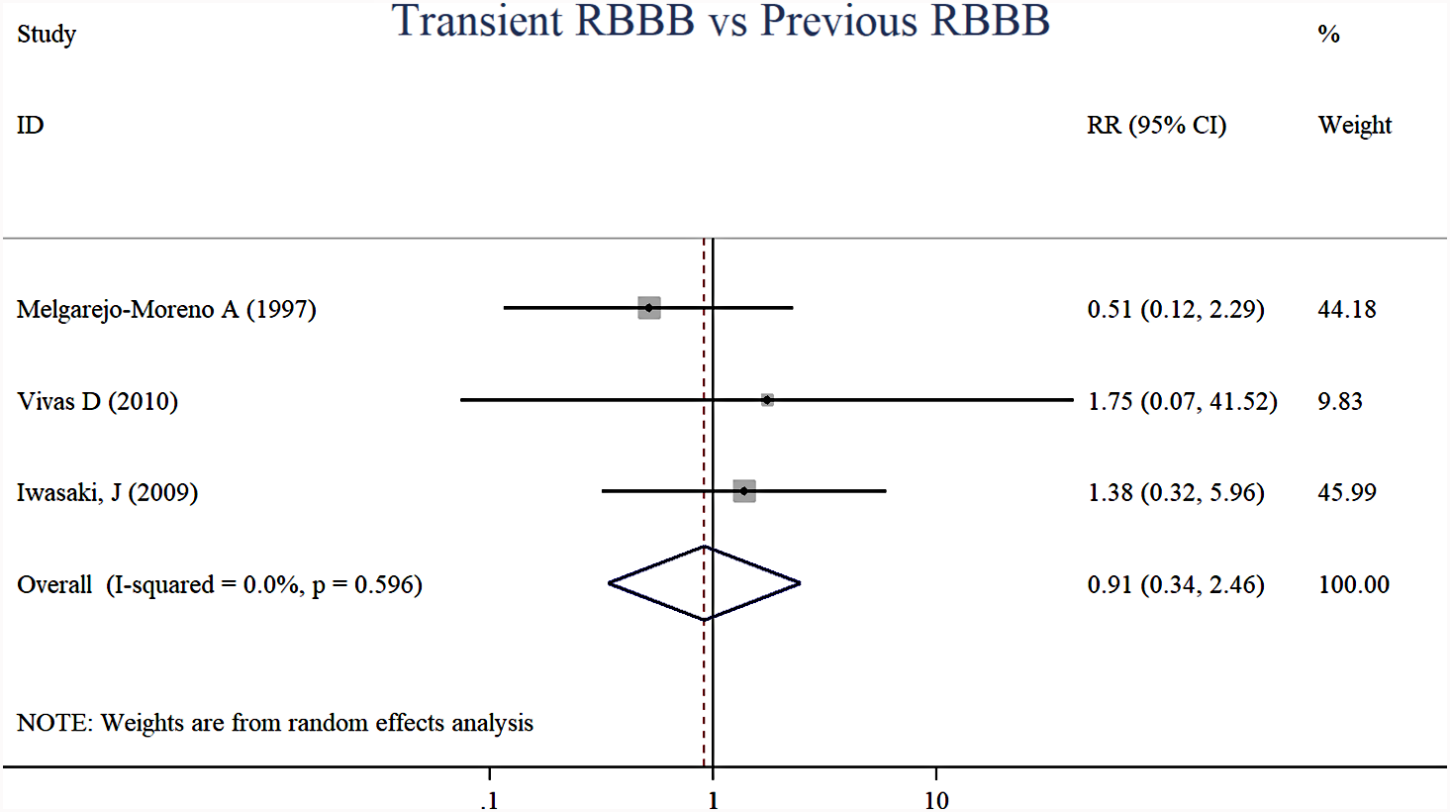
Forest plots of stratified analyses for short-time mortality (transient RBBB vs. previous RBBB).

The short-term mortality risk (RR, 0.91, 95% CI [0.34–2.46], *I*^2^ = 0.0%, *p* = 0.858, *n* = 3) of new-onset transient RBBB vs. previous RBBB in AMI patients was not different (Fig. 6) based on current evidence.

**Figure 7:**
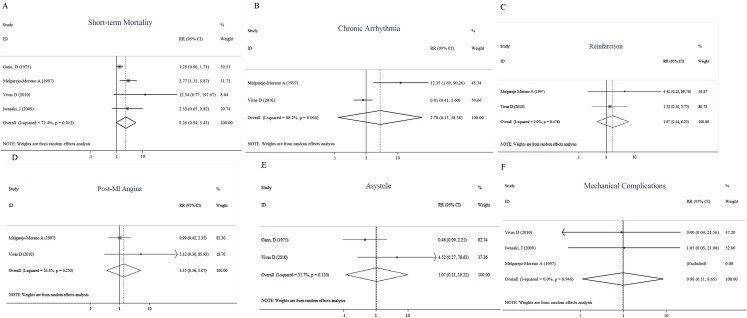
Forest plots of stratified analyses for short-time mortality (A), chronic arrhythmia (B), reinfarction (C), post-MI angina (D), asystole (E), and mechanical complication (F).

We also assessed the risks between new-onset RBBB and previous RBBB in terms of other variables. These risks included short-term mortality (RR, 2.26, 95% CI [0.94–5.43], *I*^2^ = 72.4%, *p* = 0.068, *n* = 4) (Fig. 7A) and other outcomes, like chronic arrhythmias (second-degree or higher atrioventricular block) (RR, 2.78, 95% CI [0.13–58.38], *I*^2^ = 88.2%, *p* = 0.510, *n* = 2) (Fig. 7B), reinfarction (RR, 1.67, 95% CI [0.44–6.25], *I*^2^ = 0.0%, *p* = 0.448, *n* = 2) (Fig. 7C), post-MI angina (RR, 1.35, 95% CI [0.36–5.07], *I*^2^ = 24.4%, *p* = 0.657, *n* = 2) (Fig. 7D), asystole (RR, 1.07, 95% CI [0.11–10.22], *I*^2^ = 51.7%, *p* = 0.951, *n* = 2) (Fig. 7E), and mechanical complications (RR, 0.98, 95% CI [0.11–8.65], *I*^2^ = 0.0%, *p* = 0.984, *n* = 2) (Fig. 7F). Most studies showed low to moderate heterogeneity. Specifically, reinfarction (*I*^2^ = 0.0%, *p* = 0.476), mechanical complications (*I*^2^ = 0.0%, *p* = 0.946), post-MI angina (*I*^2^ = 24.4%, *p* = 0.250), and asystole (*I*^2^ = 51.7%, *p* = 0.250) showed low to moderate insignificant heterogeneity, but short-term mortality (*I*^2^ = 72.4%, *p* = 0.012), and chronic arrhythmias (*I*^2^ = 88.2%, *p* = 0.004) showed relatively high significant heterogeneity.

Pooled RRs of sensitivity analysis (Fig. S1) did not change substantially, which indicated that most of the current results were statistically reliable.

## Discussion

This study focuses on mortality and other major cardiovascular adverse events in AMI patients with new-onset RBBB. The effects of permanent and transient new-onset RBBB are also assessed. To our knowledge, this is the first meta-analysis of observational studies on the prognostic value of new-onset RBBB in the context of AMI. In summary, we identified five studies of 874 patients and evaluated the prognostic value of new-onset RBBB in AMI. The results show that new-onset RBBB is associated with an increased long-term mortality and higher risk of ventricular arrhythmia and cardiogenic shock but not heart failure in patients with AMI. Other outcomes could not be assessed on the current data. Previous RBBB may have more relations with heart failure than new-onset RBBB in AMI patients.

Heart failure may be worse in the previous RBBB group. Based on the basic characteristics of the included studies, patients in previous RBBB group have a higher median age and more comorbidities (hypertension, diabetes mellitus, etc.), especially previous myocardial infarction and previous angina. It may explain the higher proportion of heart failure in previous RBBB group, but the accurate pathophysiological changes should be further investigated. Studies (Xiang et al., 2016; Widimsky et al., 2012) have shown that new-onset RBBBs in AMI subjects often suggest LAD lesion, proximal occlusion of coronary artery and large infarct size. It is also associated with more complications including heart failure, arrhythmias, and increasing mortality compared with non-RBBB group. Our study showed new-onset RBBB is associated with poor prognosis, including long-term mortality, ventricular arrhythmia, and cardiogenic shock in patients with AMI.

The following facts may explain the poor prognosis of new-onset RBBB in AMI. Firstly, right bundle-branch runs in interventricular septum, and the blood supply is mostly provided by the first septal branch separated from LAD. Therefore, new-onset RBBB is likely caused by proximal occlusion of LAD. New-onset RBBB in AMI is frequently caused by the complete occlusion of the infarct-related artery, the first septal branch separated from LAD or LAD per se, and sometimes even left main coronary artery (Widimsky et al., 2012). So, it is not hard to imagine that, the myocardial infarct size is likely larger in these patients (Widimsky et al., 2012). Therefore, ventricular arrhythmia and cardiogenic shock may happen frequently in AMI patients with new-onset RBBB. Secondly, previous guidelines (Steg et al., 2012; Antman et al., 2004) did not include RBBB as an indication for emergency revascularization, it might mislead clinical doctors to underestimate even ignore the diagnostic value of new-onset RBBB in AMI. Widimsky has also pointed out that even large infarcts may occur without typical STEs in AMI, thus a life-threatening AMI may be missed when ST elevation is strictly required (Widimsky et al., 2012). Clinicians should consider urgent reperfusion therapies in the setting of new-onset RBBB and ongoing ischemic symptoms. As demonstrated by our meta-analysis, there is an increasing risk of long-term mortality in patients with new-onset RBBB and AMI. Therefore, serious consequences may occur if neither urgent coronary angiography/PCI nor thrombolysis therapy is administered. So, a higher mortality can be present in AMI patients with new-onset RBBB. The guideline suggests revascularization therapies for RBBB patients with persistent ischemic symptoms, however the patients with new-onset RBBB may need more attention.

A previous study (Iwasaki et al., 2009) has indicated that, new-onset permanent RBBB is one of significant independent risk factors for predicting adverse in-hospital events. Compared with new-onset permanent RBBB in patients with AMI, the short-term mortality of new-onset transient RBBB may be different (Fig. 5), and currant evidence remains lacking. Our previous study (Jie, 2016) has shown that emergency interventional therapy could result in resolution of new-onset RBBB in AMI patients. Thus, early identification of new-onset RBBB in AMI may help clinicians to make appropriate treatment therapies and improve prognosis of patients.

LBBB masks ST-segment shifts. LBBB masks the repolarization phase changes or Q waves, while RBBB does not. However, ST depression and T inversion in precordial leads (V1–V3) can also mask minor ST elevation, therefore ST-elevation myocardial infarction (STEMI) can be missed. In other words, the presence of RBBB may also confound the diagnosis of STEMI. Thus, a number of patients with persistent ischemic symptoms and new-onset RBBB may suffer from STEMI. Therefore, we cannot ignore the diagnostic value of new-onset RBBB in AMI.

## Limitations

This study has some limitations. Since only five studies were eligible, it might lead to unavoidable bias. Some outcomes could not be assessed. It is difficult to identify the relationship between the infarct related artery and the new-onset RBBB based on the included studies. Neither long-term prognosis nor other outcomes of the two types of new-onset RBBB were analyzed. Yet, the effects of different interventions for AMI patients with new-onset RBBB were not evaluated. Larger clinical studies with rigorous designs are needed to further evaluate the prognostic value of new-onset RBBB in patients with AMI.

## Conclusion

Compared with new-onset permanent RBBB, AMI patients with new-onset transient RBBB have a lower risk of short-term mortality. Compared with those with previous RBBB, AMI patients with new-onset RBBB may have higher risks of long-term mortality, ventricular arrhythmia, and cardiogenic shock and a lower risk of heart failure. Risks of other outcomes could not be assessed based on current evidence. Revascularization therapies should be considered when persistent ischemic symptoms occur in patients with RBBB, especially with new-onset RBBB.

## Supplemental Information

10.7717/peerj.4497/supp-1 Supplemental Information 1 PRISMA checklist.

10.7717/peerj.4497/supp-2 Supplemental Information 2 Supplement. S1. The rationale for conducting the meta-analysis.
S2. The contribution that the meta-analysis makes to knowledge.

10.7717/peerj.4497/supp-3 Supplemental Information 3 Basic characteristics.

10.7717/peerj.4497/supp-4 Supplemental Information 4 New-onset RBBB vs. previous RBBB.

10.7717/peerj.4497/supp-5 Supplemental Information 5 New-onset transient RBBB vs. new-onset permanent RBBB.

10.7717/peerj.4497/supp-6 Supplemental Information 6 New-onset transient RBBB vs. previous RBBB.

10.7717/peerj.4497/supp-7 Supplemental Information 7 Sensitivity analysis.

## Abbreviations

RBB: right bundle-branch
RBBB: right bundle-branch block
AMI: acute myocardial infarction
ESC: European Society of Cardiology
CI: confidence interval
RR: relative risk
PCI: percutaneous coronary intervention
LAD: left anterior descending artery

## Additional Information and Declarations

### Data Availability

The following information was supplied regarding data availability: The raw data are provided in the Supplemental Dataset Files.
