# Supplementary material for: Prognostic value of new-onset right bundle-branch block in acute myocardial infarction patients: a systematic review and meta-analysis
Source: PeerJ. 2018 Mar 12;6:e4497. doi: 10.7717/peerj.4497 (PMC5853603; doi:10.7717/peerj.4497)
Supplement: Supplemental Information 2 — S1. The rationale for conducting the meta-analysis. S2. The contribution that the meta-analysis makes to knowledge. [file peerj-06-4497-s002.docx]

# Supplement 1. The rationale for conducting the meta-analysis

AMI patients with bundle-branch block (BBB) have worse prognoses than patients without in most previous studies. Yet investigators of these studies did not compare the effects of identified new-onset RBBB with previous RBBB (Bhalli et al., 2009; Widimsky et al., 2012). A recent systematic review (Hazem et al., 2014) showed that patients with RBBB and AMI were at more than 2-fold higher risk of all-cause mortality in 30-day follow-up compared to those without BBB. Furthermore, for patients with myocardial infarction, several other studies have reported positive associations between RBBB and all-cause mortality (Kleemann et al., 2008; Widimsky et al., 2012; Wong et al., 2006), whereas others have reported no association (Archbold et al., 1998; Juarez-Herrera & Jerjes-Sanchez, 2013).

Considering the anatomy and vascular supply of the conduction system (Mullins & Atkins, 1976; Roos & Dunning, 1978), RBBB is usually the manifestation of large infarctions that are often accompanied by heart failure, complete AV block, arrhythmias, and a high mortality (Melgarejo-Moreno et al., 1997; Klein et al., 1984). The classification of RBBB according to onset time, duration, and association with fascicular block is of clinical importance (Hindman et al., 1978; Lie et al., 1974; Ricou et al., 1991). Curiously, numbers of studies claimed that, thrombolytic treatment limits infarct size (Kloner et al., 1983; Braunwald, 1987), improves ventricular morphology and function (White et al., 1987), and decreases mortality (Yusuf et al., 1990; Grines & DeMaria, 1990; Nicod et al., 1993; 1994)**.** Moreover, some studies connected the reversibility of conduction disturbances with coronary reperfusion (Roth et al., 1993; Wiseman et al., 1989), which suggests that reperfusion therapy may prevent the appearance or limit the duration of bundle-branch blocks. Thus, it is probable that the current reperfusion therapy has changed the overall incidence and significance of RBBB in AMI. Therefore it is reasonable to reanalyze and re-realize its meaning in the reperfusion therapy era. Moreover, the new ESC guideline (Ibanez et al., 2017) recommends a primary PCI strategy should be considered when persistent ischemic symptoms occur in patients with ST-segment elevation AMI and RBBB. But, the evidence is not sufficient. The cited literature (Widimsky et al., 2012) was not clearly distinguished new-onset and unknown RBBB.

# Supplement 2. The contribution that the meta-analysis makes to knowledge

This study focuses on short-term mortality, long-term mortality and other major cardiovascular adverse events in AMI patients with new-onset RBBB. The effects of permanent and transient new-onset RBBB was also assessed. To our knowledge, this is the first meta-analysis of observational studies on the prognostic value of new-onset RBBB in the context of AMI.

**References:**

1994. Indications for fibrinolytic therapy in suspected acute myocardial infarction: collaborative overview of early mortality and major morbidity results from all randomised trials of more than 1000 patients. Fibrinolytic Therapy Trialists' (FTT) Collaborative Group. *Lancet* 343:311-322.

Archbold, R.A., Sayer, J.W., Ray, S., Wilkinson, P., Ranjadayalan, K., and Timmis, A.D. 1998. Frequency and prognostic implications of conduction defects in acute myocardial infarction since the introduction of thrombolytic therapy. *European Heart Journal* 19:893-898.

Bhalli, M.A., Khan, M.Q., Samore, N.A., and Mehreen, S. 2009. Frequency and clinical outcome in conduction defects in acute myocardial infarction. *J Ayub Med Coll Abbottabad* 21:32-37.

Braunwald, E. 1987. The path to myocardial salvage by thrombolytic therapy. *Circulation* 76:I2-I7.

Grines, C.L., and DeMaria, A.N. 1990. Optimal utilization of thrombolytic therapy for acute myocardial infarction: concepts and controversies. *Journal of the American College of Cardiology* 16:223-231.

Hazem, A., Sharma, S., Sharma, A., Leitch, C., Sharadanant, R., Uriell, M., Neutzling, K., Wang, Z., Brrionuevo Moreno, P., LeBlanc, A., Sorita, A., Vilesnki, L., Prokop, L., Makkuni, P., Ting, H.H., and Murad, M.H. 2014. Abstract 309: Is Right Bundle Branch Block Associated with Poor Outcomes in the Setting of an Acute Coronary Syndrome? A Systematic Review and Meta-analysis. *Circulation: Cardiovascular Quality and Outcomes* 7:A309.

Hindman, M.C., Wagner, G.S., JaRo, M., Atkins, J.M., Scheinman, M.M., DeSanctis, R.W., Hutter, A.J., Yeatman, L., Rubenfire, M., Pujura, C., Rubin, M., and Morris, J.J. 1978. The clinical significance of bundle branch block complicating acute myocardial infarction. 1. Clinical characteristics, hospital mortality, and one-year follow-up. *Circulation* 58:679-688.

Ibanez, B., James, S., Agewall, S., Antunes, M.J., Bucciarelli-Ducci, C., Bueno, H., Caforio, A.L.P., Crea, F., Goudevenos, J.A., Halvorsen, S., Hindricks, G., Kastrati, A., Lenzen, M.J., Prescott, E., Roffi, M., Valgimigli, M., Varenhorst, C., Vranckx, P., and Widimský, P. 2017. 2017 ESC Guidelines for the management of acute myocardial infarction in patients presenting with ST-segment elevation. *European Heart Journal*. 10.1093/eurheartj/ehx393

Juarez-Herrera, U., and Jerjes-Sanchez, C. 2013. Risk factors, therapeutic approaches, and in-hospital outcomes in Mexicans with ST-elevation acute myocardial infarction: the RENASICA II multicenter registry. *Clinical Cardiology* 36:241-248. 10.1002/clc.22107

Kleemann, T., Juenger, C., Gitt, A.K., Schiele, R., Schneider, S., Senges, J., Darius, H., and Seidl, K. 2008. Incidence and clinical impact of right bundle branch block in patients with acute myocardial infarction: ST elevation myocardial infarction versus non-ST elevation myocardial infarction. *American Heart Journal* 156:256-261. 10.1016/j.ahj.2008.03.003

Klein, R.C., Vera, Z., and Mason, D.T. 1984. Intraventricular conduction defects in acute myocardial infarction: incidence, prognosis, and therapy. *American Heart Journal* 108:1007-1013.

Kloner, R.A., Ellis, S.G., Lange, R., and Braunwald, E. 1983. Studies of experimental coronary artery reperfusion. Effects on infarct size, myocardial function, biochemistry, ultrastructure and microvascular damage. *Circulation* 68:I8-I15.

Lie, K.I., Wellens, H.J., Schuilenburg, R.M., Becker, A.E., and Durrer, D. 1974. Factors influencing prognosis of bundle branch block complicating acute antero-septal infarction. The value of his bundle recordings. *Circulation* 50:935-941.

Melgarejo-Moreno, A., Galcera-Tomas, J., Garcia-Alberola, A., Valdes-Chavarri, M., Castillo-Soria, F.J., Mira-Sanchez, E., Gil-Sanchez, J., and Allegue-Gallego, J. 1997. Incidence, clinical characteristics, and prognostic significance of right bundle-branch block in acute myocardial infarction: a study in the thrombolytic era. *Circulation* 96:1139-1144.

Mullins, C.B., and Atkins, J.M. 1976. Prognoses and management of venticular conduction blocks in acute myocardial infarction. *Mod Concepts Cardiovasc Dis* 45:129-133.

Nicod, P., Zimmermann, M., and Scherrer, U. 1993. The challenge of further reducing cardiac mortality in the thrombolytic era. *Circulation* 87:640-642.

Ricou, F., Nicod, P., Gilpin, E., Henning, H., and Ross, J.J. 1991. Influence of right bundle branch block on short- and long-term survival after acute anterior myocardial infarction. *Journal of the American College of Cardiology* 17:858-863.

Roos, J.C., and Dunning, A.J. 1978. Bundle branch block in acute myocardial infarction. *Eur J Cardiol* 6:403-424.

Roth, A., Miller, H.I., Glick, A., Barbash, G.I., and Laniado, S. 1993. Rapid resolution of new right bundle branch block in acute anterior myocardial infarction patients after thrombolytic therapy. *Pacing Clin Electrophysiol* 16:13-18.

White, H.D., Norris, R.M., Brown, M.A., Takayama, M., Maslowski, A., Bass, N.M., Ormiston, J.A., and Whitlock, T. 1987. Effect of intravenous streptokinase on left ventricular function and early survival after acute myocardial infarction. *N Engl J Med* 317:850-855. 10.1056/NEJM198710013171402

Widimsky, P., Rohac, F., Stasek, J., Kala, P., Rokyta, R., Kuzmanov, B., Jakl, M., Poloczek, M., Kanovsky, J., Bernat, I., Hlinomaz, O., Belohlavek, J., Kral, A., Mrazek, V., Grigorov, V., Djambazov, S., Petr, R., Knot, J., Bilkova, D., Fischerova, M., Vondrak, K., Maly, M., and Lorencova, A. 2012. Primary angioplasty in acute myocardial infarction with right bundle branch block: should new onset right bundle branch block be added to future guidelines as an indication for reperfusion therapy? *European Heart Journal* 33:86-95. 10.1093/eurheartj/ehr291

Wiseman, A., Ohman, E.M., and Wharton, J.M. 1989. Transient reversal of bifascicular block during acute myocardial infarction with reperfusion therapy: a word of caution. *American Heart Journal* 117:1381-1383.

Wong, C.K., Stewart, R.A., Gao, W., French, J.K., Raffel, C., and White, H.D. 2006. Prognostic differences between different types of bundle branch block during the early phase of acute myocardial infarction: insights from the Hirulog and Early Reperfusion or Occlusion (HERO)-2 trial. *European Heart Journal* 27:21-28. 10.1093/eurheartj/ehi622

Yusuf, S., Sleight, P., Held, P., and McMahon, S. 1990. Routine medical management of acute myocardial infarction. Lessons from overviews of recent randomized controlled trials. *Circulation* 82:I117-I134.
