# Supplementary figures and images for: Prognostic value of new-onset right bundle-branch block in acute myocardial infarction patients: a systematic review and meta-analysis
Source: PeerJ. 2018 Mar 12;6:e4497. doi: 10.7717/peerj.4497 (PMC5853603; doi:10.7717/peerj.4497)

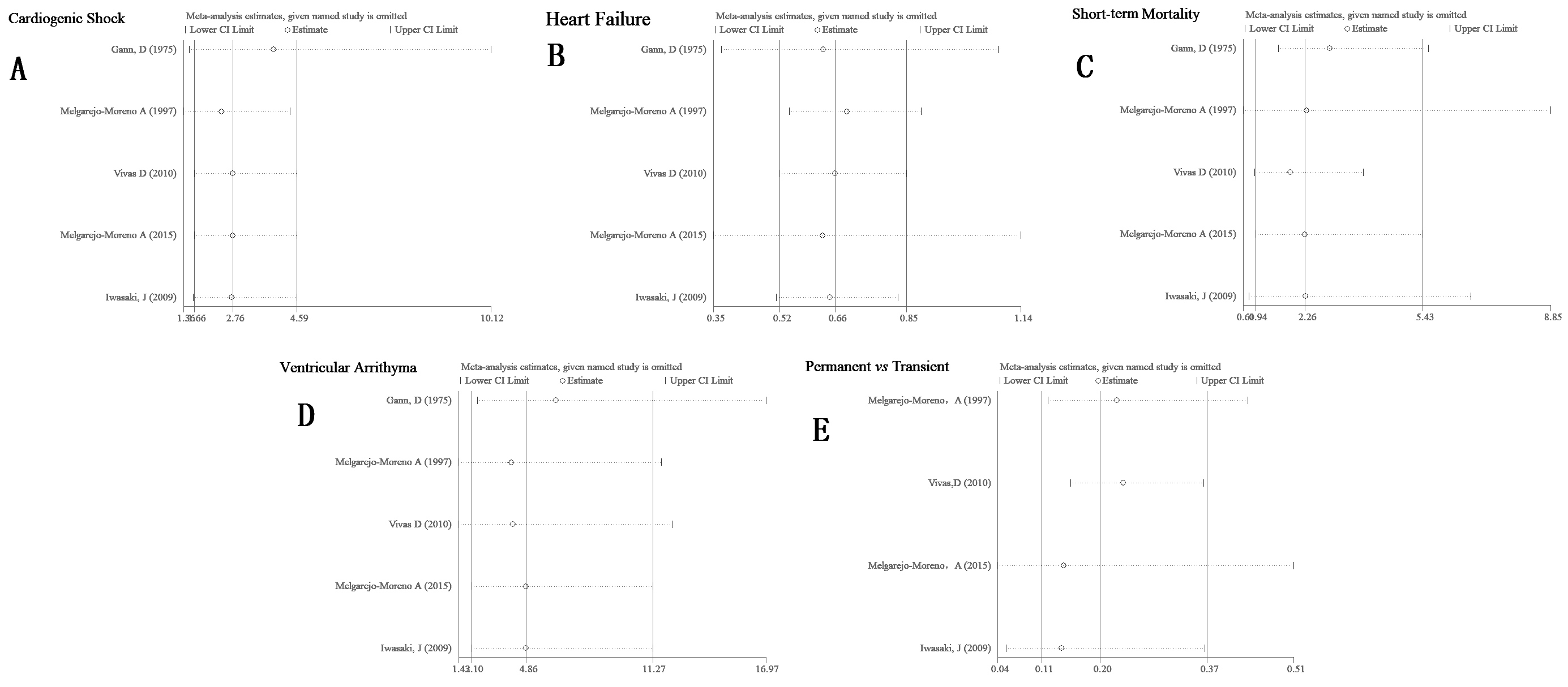

Supplement: Supplemental Information 7 [file peerj-06-4497-s007.png]
